# Differential Expression of Novel Potential Regulators in Hematopoietic Stem Cells 

**DOI:** 10.1371/journal.pgen.0010028

**Published:** 2005-09-02

**Authors:** E. Camilla Forsberg, Susan S Prohaska, Sol Katzman, Garrett C Heffner, Josh M Stuart, Irving L Weissman

**Affiliations:** 1 Departments of Pathology and Developmental Biology, Institute of Cancer and Stem Cell Biology and Medicine, Stanford University Medical School, Stanford, California, United States of America; 2 Biomolecular Engineering, University of California at Santa Cruz, Santa Cruz, California, United States of America; The Jackson Laboratory, United States of America

## Abstract

The hematopoietic system is an invaluable model both for understanding basic developmental biology and for developing clinically relevant cell therapies. Using highly purified cells and rigorous microarray analysis we have compared the expression pattern of three of the most primitive hematopoietic subpopulations in adult mouse bone marrow: long-term hematopoietic stem cells (HSC), short-term HSC, and multipotent progenitors. All three populations are capable of differentiating into a spectrum of mature blood cells, but differ in their self-renewal and proliferative capacity. We identified numerous novel potential regulators of HSC self-renewal and proliferation that were differentially expressed between these closely related cell populations. Many of the differentially expressed transcripts fit into pathways and protein complexes not previously identified in HSC, providing evidence for new HSC regulatory units. Extending these observations to the protein level, we demonstrate expression of several of the corresponding proteins, which provide novel surface markers for HSC. We discuss the implications of our findings for HSC biology. In particular, our data suggest that cell–cell and cell–matrix interactions are major regulators of long-term HSC, and that HSC themselves play important roles in regulating their immediate microenvironment.

## Synopsis

Hematopoietic, or blood-forming, stem cells (HSC) are responsible for the continual replenishment of all blood cells throughout life. This ability to both renew themselves and give rise to expanded populations of differentiating and mature cells is a hallmark of stem cells and is therefore an area of intense research. The rarity of HSC as well as their location in the bone marrow environment has made it difficult to identify the genes that regulate these properties. The earliest stages of blood development begins with the long-term (LT) repopulating HSC that then differentiate into short-term (ST) repopulating HSC and non-self renewing multipotent progenitors (MPP). The authors investigated the gene expression differences in these highly purified populations that differ mainly in their capacity to self renew, and identified a number of genes specific to each of these populations. Intriguingly, many of these genes code for proteins that are involved in cell–cell and cell–matrix interactions that were not previously identified on these populations. These novel discoveries will, together with future experiments, enhance our understanding of the basic biology of stem cells and their clinical uses.

## Introduction

Mature blood cells have a high turnover rate and need to be constantly replaced as well as respond to more acute conditions such as blood loss or infections, requiring the rapid generation of millions of new blood cells. This demand is fulfilled for life by a pool of hematopoietic stem cells (HSC). The long-term repopulating HSC (LT-HSC) thus has to be capable of differentiating without depleting the stem cell pool, thereby satisfying the definition of a stem cell: the ability at the single cell level to both self-renew and differentiate into more mature cell types. LT-HSC normally reside in the bone marrow and have essentially six developmental choices: remain quiescent, differentiate, self-renew, migrate, enter senescence, or undergo apoptosis. Such fate decisions are likely controlled both by HSC-intrinsic mechanisms and by the bone marrow microenvironment or “niche.” It has proved difficult to define the complex intrinsic and extrinsic mechanisms that govern the balance of these decisions. In hematopoiesis, only LT-HSC are capable of lifelong self-renewal and, therefore, is the operative population in hematopoietic transplantation. Understanding how HSC fate decisions are controlled is therefore of critical importance.

The expression profiles of primitive hematopoietic cells defined by various criteria have previously been compared to other hematopoietic and non-hematopoietic cell types [1–5]. In addition, several molecules and pathways have been implicated in HSC self-renewal, including HoxB4, Bmi1, the Wnt/β-catenin signaling pathway, and Notch [6–9]. As the bone marrow microenvironment likely provides unique cues necessary for proper HSC function, cell surface proteins mediating these signals should play important roles in HSC fate decisions. While there have been recent advances in defining a potential HSC niche within the bone marrow [10,11], little is known about the specific signals regulating HSC in vivo. A central question is how the interplay of soluble ligands, matrix interactions, and cell–cell contacts influence HSC fate. Recent evidence points to a role for the angiopoietin receptor Tek, also known as Tie2, in maintaining transplantable HSC [12]. Likewise, HSC in mice null for Mmp9, a matrix metalloproteinase (Mmp) that facilitates cell migration by proteolytic cleavage, have impaired proliferation and differentiation capabilities [13]. In addition, mice lacking integrin β1, part of the HSC homing receptor α4β1 [14], cannot establish fetal liver hematopoiesis [15], presumably due to a failure of LT-HSC to engraft at this fetal site. Thus, there is increasing evidence that interactions with the environment are important for the maintenance of HSC self-renewal capability. A thorough understanding of cell surface molecules expressed on HSC is an important step in identifying functional interactions with the environment.

Here, we have carefully analyzed the transcription profiles of three highly purified subpopulations within the mouse adult bone marrow lineage^−^/c-kit^+^/Sca1^+^ (KLS) fraction: LT-HSC (defined as Thy1.1^lo^/Flk2^−^ KLS), short-term (ST)-HSC (Thy1.1^lo^/Flk2^+^ KLS), and multipotent progenitors (MPP) (Thy1.1^−^/Flk2^+^ KLS) [16]. These three populations have the ability to give rise to both lymphoid and myeloid lineages [16] and platelets (E. C. F., E. Passegué, and I. L. W., unpublished data) when transplanted into irradiated mice. Thus, LT-HSC, ST-HSC and MPP have similar multilineage potential, but differ in their self-renewal and proliferative capacity. All long-term repopulating activity is contained in the LT-HSC fraction; thus, cells within this fraction are the only cells capable of maintaining hematopoiesis for the life of the host. As LT-HSC differentiate to ST-HSC and then to MPP, self-renewal capability progressively declines [16]. Therefore, cells derived from transplanted ST-HSC and MPP decrease to undetectable levels in peripheral blood by about ten weeks posttransplant. LT-HSC, ST-HSC and MPP also differ in their cell cycle status. While most LT-HSC are quiescent, a larger fraction of ST-HSC and MPP are in cycle under steady-state conditions (E. Passegué, A. J. Wagers, and I. L. W., unpublished data). Based on these small, but functionally critical differences, we reasoned that comparing the transcriptional profile of these three populations would reveal genes specifically involved in self-renewal, quiescence, and proliferation mechanisms. Here, we present new data on selective gene expression in highly purified HSC subsets and discuss the possible implications for HSC biology.

## Results

### Experimental Design and Data Analysis

**Figure 1:**
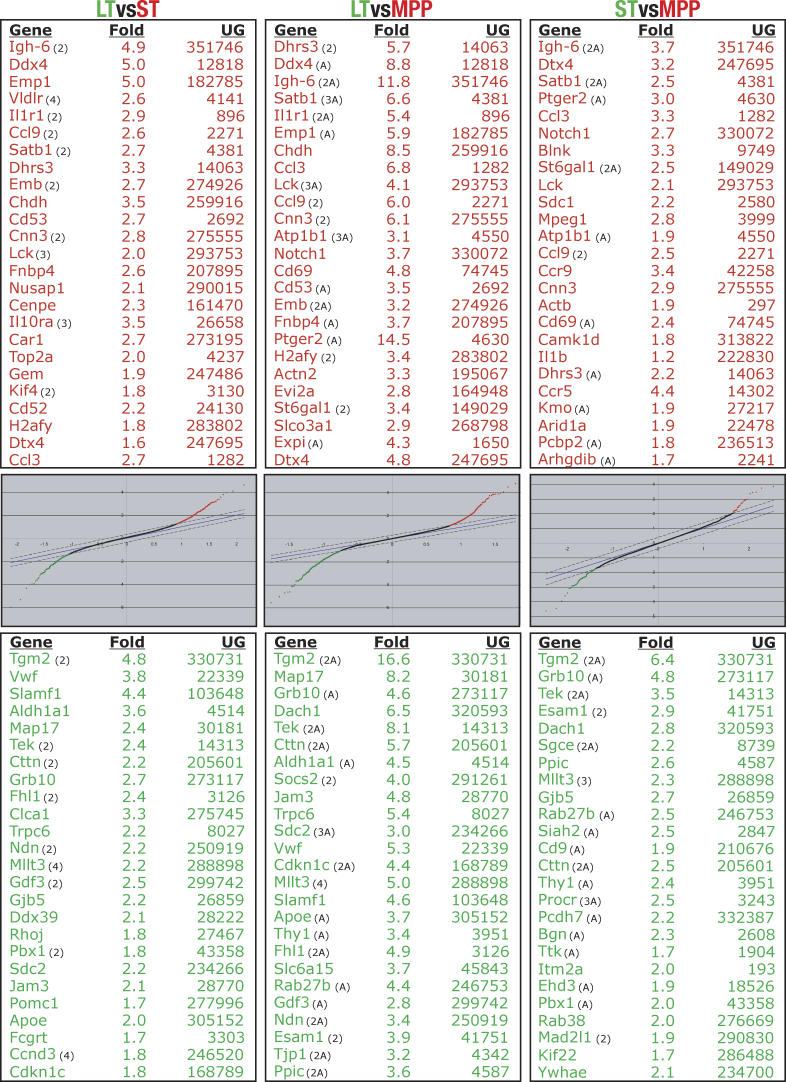
Top 25 Differentially Regulated Transcripts with Corresponding SAM Plots for Each Comparison Only known, unique genes are listed; thus, ESTs were removed, and genes appearing more than once in the same list are denoted with number of appearances in parentheses. An “A” in parenthesis indicates that results from analogous experiments using Agilent arrays are consistent with the Stanford Microarray Database array data. The LT-HSC to ST-HSC comparison was not performed with Agilent arrays.

**Figure 2:**
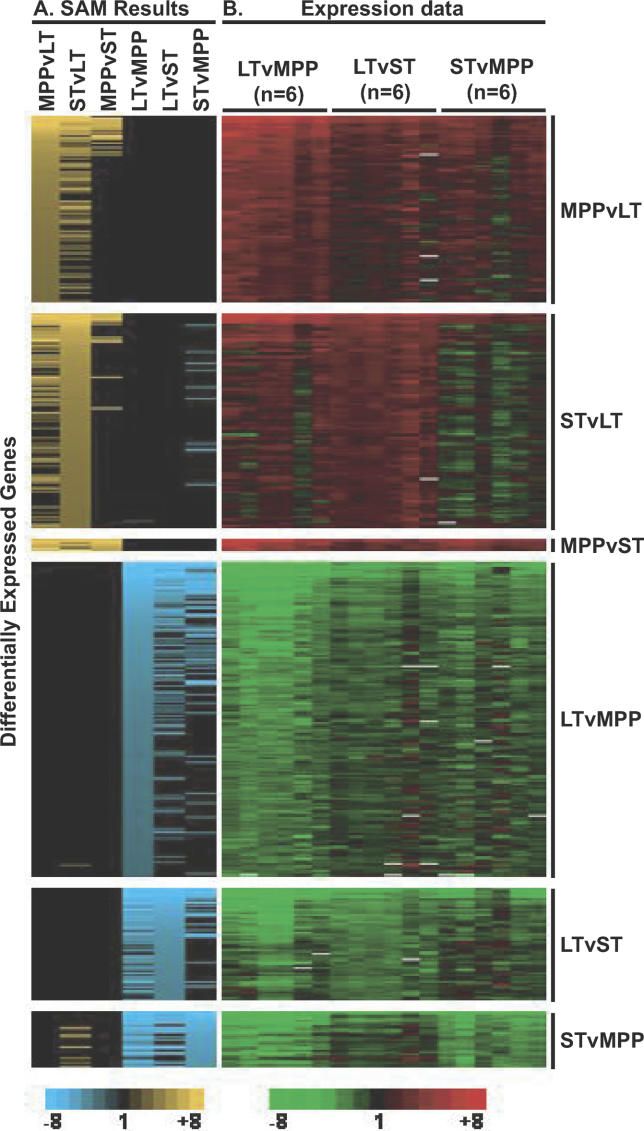
Heat Map Representation of Differentially Regulated Transcripts Rows represent genes and columns represent array comparisons between cell populations as indicated. (A) Significance score by SAM is indicated from top to bottom of each comparison (*n* = 6) by gradients of yellow or blue, with brighter yellow or blue indicating higher significance. Yellow or blue in additional columns indicate that the gene was also differentially expressed between these cell types (e.g., many of the genes upregulated in MPP versus LT-HSC were also upregulated in ST-HSC compared to LT-HSC). (B) Conventional red-green expression data for all the differentially regulated genes for each array. Red indicates genes upregulated in the more differentiated cell population (e.g., upregulated in MPP when compared to LT-HSC); green indicates upregulated in the less differentiated cell population.

To obtain accurate and reliable transcription profiles of LT-HSC, ST-HSC and MPP, we isolated highly pure, functionally defined cell populations and performed pairwise competitive hybridizations in three independent experiments, each with a “dye swap,” so that each comparison was performed six times. This allowed us to identify small but reproducible changes in gene expression. We used Stanford microarrays spotted with about 42,000 cDNAs and expressed sequence tags (ESTs) from a wide variety of tissue- and developmental stage-specific libraries, including a library made from bone marrow-subtracted HSC cDNA [4] and a selection of genes involved in development and hematopoiesis. We employed stringent criteria when evaluating and scoring genes as differentially expressed. This resulted in six gene lists (two for each comparison; Figure 1 and Tables S1−S6). As expected, most of the differentially regulated transcripts came from the comparison of LT-HSC to MPP, with 527 transcripts upregulated in LT-HSC and 323 transcripts upregulated in MPP, totaling 850 differentially regulated transcripts. Between LT-HSC and ST-HSC 565 transcripts were differentially regulated, as were 111 transcripts between ST-HSC and MPP (Table 1). The top 25 statistically significant up- and downregulated genes from each comparison are listed in Figure 1. Complete lists of all differentially regulated genes, including ESTs and transcripts represented by multiple spots, are presented in Tables S1−S6 and summarized as a heat map representation in Figure 2.

**Table 1:**
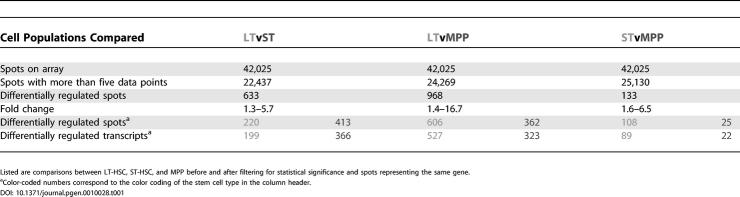
Summary of the Numbers of Differentially Regulated Transcripts

**Figure 3:**
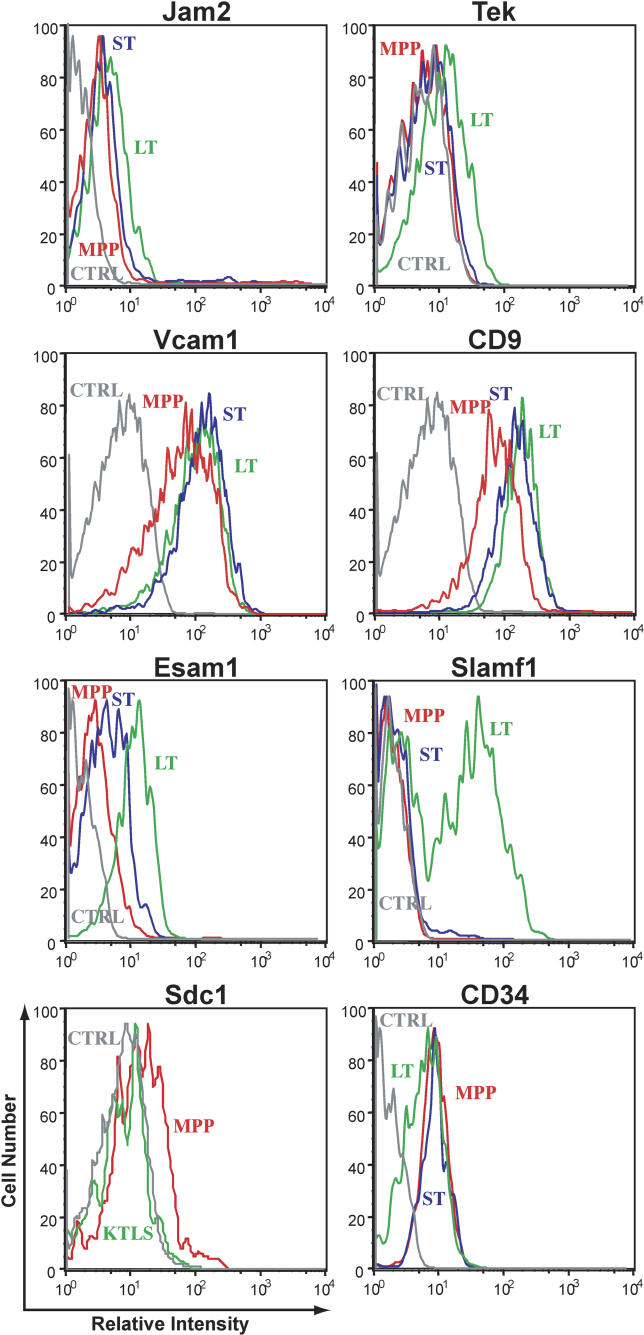
Flow Cytometric Analysis of Cell Surface Protein Levels on LT-HSC, ST-HSC, and MPP Relative to Control Green represents LT-HSC, blue represents ST-HSC, red represents MPP, and grey represents the control.

The reliability of the resulting lists of differentially expressed genes was then assessed in several ways. First, as illustrated in Figure 2, the reproducibility between and across arrays is extremely good and consistent with the previously described linear relationship between these populations, where LT-HSC give rise to, first, ST-HSC, which in turn differentiate into MPP. Second, these data accurately reflect the expression of several transcripts known to be differentially expressed in hematopoietic stem and progenitor cells. These include the sort markers Thy1.1 (upregulated in LT-HSC) and Flk2 (upregulated in MPP). The transporter Abcg2, responsible for the “side population” phenotype [17], is upregulated in LT-HSC, while CD34 transcripts increase with differentiation, consistent with CD34 protein levels in previous studies [18]. A fraction of LT-HSC is negative for CD34 cell surface protein, while the vast majority of ST-HSC and MPP are CD34-positive (Figure 3). Aldehyde dehydrogenase, Aldh1a1, and Tek transcript levels are also higher in LT-HSC in agreement with their selective expression by HSC reported previously [12,19,20]. In addition, numerous transcripts scored consistent with each other on the Stanford cDNA arrays if represented by two or more spots, and when assayed using Agilent oligonucleotide arrays (see Figure 1; unpublished data). We also confirmed the expression patterns of several of the transcripts by quantitative RT-PCR of unamplified RNA from double-sorted cells (Figure S1) and by flow cytometry of protein levels (Figure 3). In no case did RT-PCR or cell surface protein levels contradict the array data. Thus, we believe that these analyses accurately reflect the true transcription profile of these three populations.

To detect potential self-renewal or proliferation loci we determined the chromosomal location of genes differentially regulated between LT-HSC and MPP (Figure S2). The most striking difference was observed on the X chromosome, where 23 genes upregulated in LT-HSC but only one MPP-specific gene reside. The highest enrichment of MPP-specific genes was a 2.4-fold increase on chromosome 19. The 23 LT-specific genes are listed in Table S7. We detected no enrichment of LT-HSC-specific genes on chromosome 17, which we and others previously showed to contain a stem cell frequency locus [2,21].

**Figure 4:**
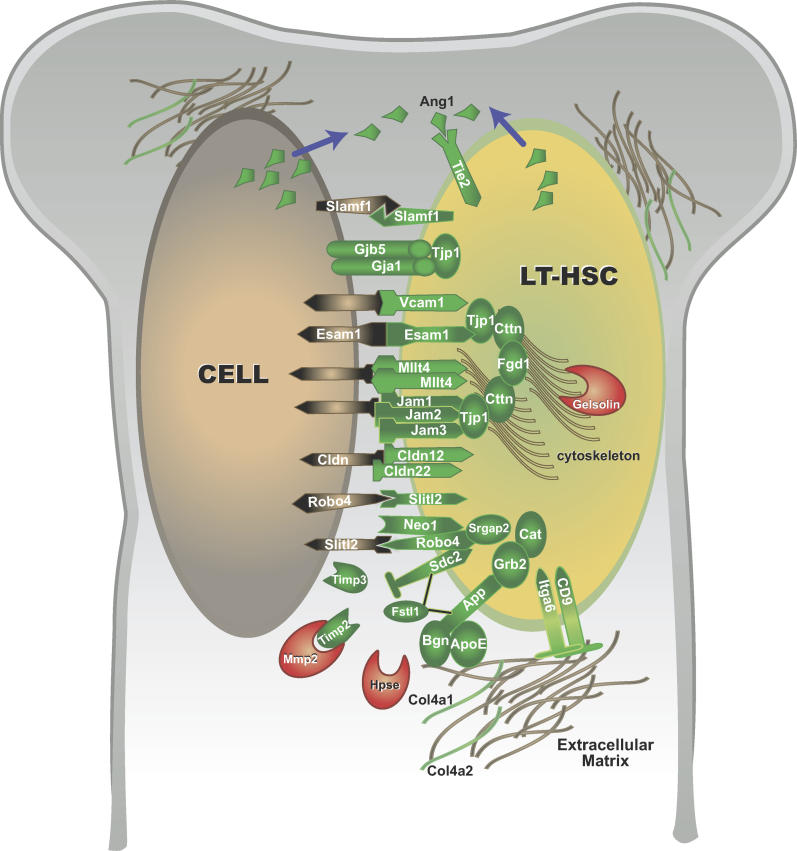
Hypothetical Model of Selected Potential Interactions of Proteins Corresponding to Differentially Expressed Transcripts Molecules upregulated in LT-HSC are in green; those upregulated in MPP are red. Depicted protein interactions and colocalization are based on published reports in various mammalian and nonmammalian systems. Single cells expressing all the proteins as depicted in this cartoon may not exist. Not drawn to scale.

To assess objectively what types of genes were differentially regulated, we used the gene classification tool Gene Ontology (GO) [22]. GO has classified a large number of genes based on biological processes, molecular function, and cellular component. Genes enriched in the more primitive cells are overrepresented in the GO categories involving cell junction, anion transport, extracellular space, and oxidoreductase activity, whereas cell differentiation enriched for transcripts in the cell cycle, immune response, taxis/chemotaxis, and regulation of transcription categories. However, as only a fraction of the genes in our data set has been classified by GO, the majority were broadly categorized by reviewing published literature and discussed based on cellular location moving from the extracellular space inward. Genes discussed in the text are summarized in Table 2 and selected transcripts are depicted in Figure 4. Additional references are presented in Table S8.

**Table 2:**
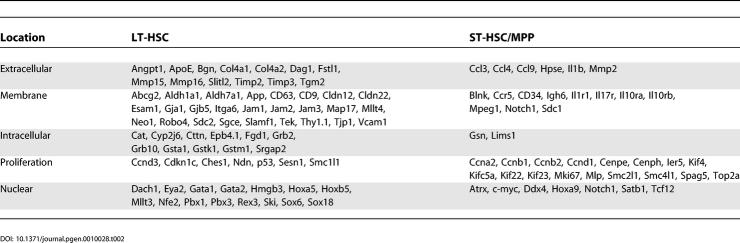
Differentially Regulated Transcripts Discussed in the Text

### Extracellular Matrix and Other Secreted Proteins

The extracellular matrix (ECM) proteins upregulated in LT-HSC include transglutaminase2 (Tgm2), biglycan, Dag1/dystroglycan, ApoE, Emilin1, Calsyntenin1, collagens type 4a1 and 4a2, and the heparin-binding protein Fstl1. Tgm2 is a widely expressed protein cross-linking enzyme that can function in the ECM, cytosol, and nucleus. The proteoglycan biglycan is highly expressed in connective tissues including bone, and mice lacking biglycan are prone to osteoporosis. ApoE, an ECM protein and a component of low-density lipoproteins, interacts directly with biglycan, and both ApoE and biglycan bind to membrane proteins preferentially expressed by LT-HSC (see below). By expressing genes encoding structural components of the ECM such as collagens and proteoglycans, LT-HSC may contribute to the architecture of its immediate microenvironment. In contrast, matrix metalloproteinases (Mmp) facilitate cell motility and are important for HSC differentiation [13]. Mmp15 and Mmp16 are upregulated in LT-HSC, but their activity may be inhibited by high levels of the Mmp inhibitors Timp2 and Timp3. Interestingly, concurrent with Timp2 downregulation in MPP, the main target of Timp2, Mmp2, is upregulated. Another facilitator of cell motility, heparanase, is also upregulated in MPP. Heparan sulfate-bearing membrane receptors, potential substrates for heparanase, are upregulated in LT-HSC (below). Together, the increases in heparanase and Mmp2 expression and decreases in Timp2 and Timp3 levels may act to destabilize cell interactions with the matrix and allow a subset of HSC to exit the niche and undergo differentiation.

### Membrane, Cell Junction, and Adhesion Molecules

The “cell junction” GO category includes a number of genes not previously implicated in HSC biology. Transcripts upregulated in LT-HSC in this category include the junction adhesion molecules Jam1/F11R, Jam2 and Jam3, Claudin12, Claudin22, the tight junction protein Tjp1/ZO-1, and the connexins Gja1 and Gjb5. Jam2 protein is detectable on the surface of LT-HSC, and its levels decrease as LT-HSC differentiate into ST-HSC and MPP (see Figure 3). Jams, Tjps, and claudins commonly colocalize in epithelial cells to form tight junctions. Tjp1 is also found at gap junctions with the channel-forming connexins. The differential expression of two connexins in LT-HSC, Gja1 and Gjb5, is consistent with evidence for gap junctions functioning in stem cell regulation in human ES cells [23], and in *Drosophila,* where the gap junction protein Zpg is necessary for proper germ stem cell differentiation [24]. It will be interesting to determine whether these proteins form functional channels in HSC, and if so, with what partner cells and what types of molecules are exchanged between cells.

VCAM1 and ESAM1 are related adhesion molecules upregulated in LT-HSC both at the transcript and protein level (see Figure 3). VCAM1 interaction with integrin α4β1 mediates cell–cell interactions in multiple cell types, and both VCAM1 and integrin α4β1 have been implicated in HSC homing to the bone marrow. As at least a subset of LT-HSC express α4β1 [14], detection of VCAM1 protein on all LT-HSC (Figure 3) indicates that a single LT-HSC can express both α4β1 and VCAM1. ESAM1 was first described on endothelial cells, but is also expressed on megakaryocytes and platelets [25,26]. ESAM1 mediates homophilic interactions between endothelial cells and colocalizes with tight junction proteins and claudins. The self-ligand Slamf1, known for its roles in bidirectional T- and B-cell stimulation, is upregulated in LT-HSC, as shown here and in other studies (S. Morrison, personal communication). Interestingly, the cell surface protein profile divides LT-HSC into Slamf1 positive and negative fractions, whereas ST-HSC and MPP are uniformly Slamf1 negative (Figure 3). As HSC negative for CD34 are Slamf1 positive, and there is an inverse relationship between Slamf1 and Flk2 surface expression on both HSC and myeloid progenitor populations, Slamf1 functions as a useful marker to separate these populations (D. Bryder, E. C. F., and I. L. W., unpublished data). Ongoing studies indicate that there are functional differences between Slamf1 positive and negative stem and progenitor populations.

LT-HSC also express higher levels of the receptor Robo4, its putative ligand Slit-like2, and the effector protein Srgap2 than do ST-HSC or MPP. In *Drosophila*, Slit/Roundabout signaling promotes asymmetric division, and it inhibits cell migration in both *Drosophila* and mammalian systems [27,28], perhaps by inhibition of Mmp2 and Mmp9 by Slit [29]. Robo4 can interact directly with DCC, a homolog of Neogenin which is also upregulated in LT-HSC over MPP. In other systems, Neogenin and DCC are implicated in cell adhesion, polarity, and migration, and are receptors for the Netrin family of chemoattractants. Interestingly, Slit and Netrin signaling influence each other [30] and can have opposing effects [31]. Slit signaling can also modulate Cxcr4-mediated chemotaxis [29], and may therefore play a role in HSC migration toward Sdf1 gradients [32,33].

*Drosophila* Roundabout binds directly to Syndecan [27], and Roundabout mutants phenocopy the Syndecan mutant [34]. The syndecans are transmembrane heparan sulfate-decorated molecules that link the cytoskeleton to the extracellular space and participate in cell–cell and cell–matrix interactions, cell proliferation, and growth factor signaling. We found Sdc1 transcripts upregulated in MPP, and a fraction of MPP also expresses the protein on the cell surface (Figure 3). In contrast, Sdc2 transcripts are enriched in LT-HSC. Both Sdc1 and Sdc2 are coreceptors for FGF and GM-CSF [35,36], molecules with known functions in hematopoiesis. Syndecan-mediated adhesion is regulated by metalloproteinases and their inhibitors as well as by heparanase. Cleavage of the heparin chains by heparanase or proteolysis by metalloproteinases increases cell migration, the latter of which can be inhibited by Mmp inhibitors such as Timp3 [27]. Thus, differential expression of syndecans and regulation of their cell–cell and cell–matrix interactions may control retention versus release of cells from the niche.

Amyloid beta precursor protein (App) is a heparin-binding cell adhesion molecule that interacts with two of the extracellular matrix molecules upregulated in LT-HSC, ApoE [37] and biglycan [38]. Mutations of App have been implicated in Alzheimer's disease. Proteolytic cleavage of App by γ-secretase and presenilin leads to nuclear translocation and transcriptional activation. App may thus directly transmit extracellular signals to regulate transcription of specific target genes in LT-HSC.

Tek is expressed by HSC [12,19], and a role for Tek in maintaining HSC quiescence through interaction with the niche was suggested [12]. Tek transcript levels are indeed higher in LT-HSC than in ST-HSC or MPP, and all detectable cell surface expression of Tek protein is contained within the LT-HSC fraction (Figure 3). We and others [1] also detected robust and differential transcript levels of the Tek ligand angiopoietin1 in LT-HSC, suggesting potential autocrine or paracrine Tek activation.

Several other proteins corresponding to transcripts enriched in LT-HSC might mediate cell–cell and cell–matrix interactions. Map17 is expressed mainly in the kidney but is also enriched in LT-HSC over MPP. Although upregulated in carcinomas, overexpression of Map17 in colon carcinoma cells can inhibit cell proliferation and tumor growth [39]. The tetraspanins CD9 and CD63 can function as metastasis suppressors, potentially due to association with integrins [40]. The surface protein levels of CD9 correlate with the transcript profile (Figure 3). Elsewhere we have shown that CD9 is a marker for the megakaryocyte progenitor, MKP [41]. CD9 can interact with integrin α6 (Itga6) [42–44], which, together with integrin β1, form a cell surface laminin-binding receptor. Cell surface expression of Itga6 and Itgb1 on HSC was demonstrated previously [14]. Itga6 may be a general stem cell marker since it is upregulated on LT-HSC in addition to stem cells of several other tissues [45,46]. Another surface protein upregulated in LT-HSC, Mllt4, is an actin filament-binding protein that belongs to a cadherin/catenin adhesion system with roles in the organization of homotypic and heterotypic cell–cell adherens junctions [47].

In contrast to the abundance of cell adhesion molecules upregulated in LT-HSC, MPP express a number of cell surface receptors implicated in immune response and chemokine activation. These include IL10rα and β, IL1r1, IL17r, Mpeg1, Blnk, Notch1, Ccr5, and Igh6. These and other receptors selectively expressed by MPP can render cells responsive to chemotactic and inflammatory response molecules such as Ccl3, Ccl4, Ccl9, and IL1β, whose transcripts are also upregulated in MPP. It is possible that distinct subsets of MPP express high levels of particular transcripts as an early indicator of commitment to different downstream lymphoid and myeloid lineages, or that the presence of both types of transcripts are indicative of promiscuous expression associated with lineage commitment [48,49].

### Intracellular Adaptor and Signaling Molecules

Links between the extracellular space and the actin cytoskeleton are provided by several transcripts preferentially expressed by LT-HSC. This includes direct interaction with actins by transmembrane proteins such as Mllt4 and sarcoglycan E, as well as by use of adaptor molecules such as the erythrocyte cell shaping protein Epb4.1, the Slit/Robo effector protein Srgap2, cortactin, and Fgd1. Cortactin functions in cell shape, actin organization, and cell adhesion by interacting directly with actin and with components of adherens-type junctions, including cadherins, catenins, and Tjp1 [50]. Cortactin also interacts with Fgd1 [51], a protein with roles in actin organization and cell shape.

The growth factor receptor-bound adaptor proteins Grb2 and Grb10 are also upregulated in LT-HSC. In other systems, Grb10 regulates signaling from several receptors, including IGFR, PDGFR, EGFR, and VEGFR2, at least in part by antagonizing receptor degradation by Nedd4 [52], a ubiquitin ligase robustly expressed in HSC. Depending on the context, Grb10 may promote or inhibit cell growth [53]. Grb2 can interact with App to regulate apoptosis in a Map kinase-dependent manner in neuroblastoma cells [54]. Interestingly, Grb2 also binds catalase [55], an enzyme recently implicated in HSC self-renewal [56]. Catalase is upregulated in LT-HSC together with other transcripts implicated in oxidoreductase activity such as Sestrin1, the known stem cell marker aldehyde dehydrogenase Aldh1a1 and its homolog Aldh7a1, the cytochrome P450 Cyp2j6, and glutathione-S-transferases Gsta1, Gstk1, and Gstm1. These enzymes may render LT-HSC uniquely resistant to stress, a property that would be particularly important for long-lived cells such as stem cells.

MPP have higher transcript levels for proteins with potentially opposite effects of those upregulated in LT-HSC. Two such examples that regulate cell growth, proliferation, and differentiation are Gelsolin, a calcium-dependent actin-depolymerizing protein, and Lims1, an adaptor protein mediating integrin and growth factor signaling at focal adhesions [57,58].

### Proliferation and Cell Cycle Genes

The proliferative activity of LT-HSC increases as they differentiate into ST-HSC and MPP (E. Passegué, A. J. Wagers, and I. L. W., unpublished data). Thus, we expected transcripts related to cell cycle control to be differentially regulated. Indeed, the most significantly enriched GO categories for MPP are related to cell proliferation. The array data presented here are corroborated by the qRT-PCR data performed on the same cell populations (E. Passegué, A. J. Wagers, and I. L. W., unpublished data) and indicate that LT-HSC express higher levels of cyclin D3 and the cyclin-dependent kinase inhibitor p57/Cdkn1c, whereas ST-HSC/MPP express higher levels of cyclins A2, B1, B2, and D1 and the proliferation marker Ki67. Our array analysis identified several additional transcripts that may play a role in regulating HSC cell cycle status. Transcripts enriched in LT-HSC include p53, the growth suppressor necdin; Sesn1, a regulator of cell cycle arrest and a negative regulator of cell proliferation; and Ches1, a probable transcriptional activator that may be involved in DNA damage-induced cell cycle arrests.

Ier5, a mediator of mitogenic signals, is upregulated in ST-HSC and MPP, as are several transcripts associated with mitotic chromosomes. These include Spag5, the centromere proteins Cenph and Cenpe, and the kinesins Kif4, Kifc5a, Kif22, and Kif23. Marcks-like protein (Mlp), a component of the anaphase-promoting complex, and the topoisomerase Top2a are also increased. Two cohesin-complex transcripts, Smc2l1 and Smc4l1, are upregulated as LT-HSC differentiate, potentially to promote chromosome segregation during the increased cycling of ST-HSC and MPP. In contrast, Smc1l1 is upregulated in LT-HSC. Smc1l1 is phosphorylated by ataxia telangiectasia mutated, indicating a potential role for this protein in DNA repair. This is also of note, since ataxia telangiectasia mutated-mediated responses to oxidative stress recently were implicated in HSC self-renewal [56]. Although several of the transcripts in this category may be upregulated as a consequence of increased cycling, some likely have more causal roles. Proliferation is likely regulated to a significant extent by high expression of cell cycle inhibitors such as p57/Cdkn1c in LT-HSC. However, of note is that cyclin D3 is upregulated in LT-HSC, and it will be interesting to determine whether cyclin D3 activity favors self-renewing divisions over divisions associated with differentiation.

### Transcription Factors and Other Nuclear Proteins

Transcriptional regulators with higher expression in LT-HSC include the erythroid- and megakaryocyte-specific transcription factors Gata1, Gata2, and Nfe2; members of the Hox family of transcription factors, Hoxa5 and Hoxb5; the Hox-interacting Pbx1 and Pbx3; the high-mobility group proteins Sox6, Sox18, and Hmgb3; and Ski, Dachshund1, and Eya2. Ski and Dachshund belong to the Ski/Sno family of proto-oncogenes implicated in TGF-β/Smad signaling. They generally act as corepressors of transcription in concert with DNA-binding proteins and histone deacetylases. Ski has previously been shown to be expressed in hematopoietic cells [59] and to play a role in regulating cell proliferation by interfering with TGF-β signaling [60] or Myb-mediated transcriptional activation [61]. Indeed, as transcript levels of Ski decline in MPP, there is increased expression of the Myb target gene *c-myc*. This transcriptional repressor or activator depending on the context, might affect the balance of HSC self-renewal, as mice lacking hematopoietic expression of c-myc have increased numbers of phenotypic HSC and fewer differentiated progeny [62].

Dachshund proteins operate together with the Eya family of proteins to regulate gene expression. While Dach can repress transcription either by binding directly to DNA or as a corepressor for the Six family of proteins, Eya factors are transcriptional coactivators [63,64]. Both Eya1 and Eya2 transcripts are enriched in HSC when compared to hematopoietic progenitor populations [4], but only Eya2 is differentially expressed among LT-HSC, ST-HSC and MPP. The Eya proteins interact with G proteins in the cytosol, translocate to the nucleus, and use their phosphatase activity to regulate transcription [65]. The Eya/Dach complex has been implicated in modulating precursor cell proliferation [65] by regulating transcription of cell cycle inhibitors such as p27/Cdkn1b [63].

The high-mobility group proteins Hmgb3, Sox6, and Sox18 are upregulated in LT-HSC compared to MPP. Sox18 has been implicated in vascular development and transactivates the Vcam1 promoter [66], consistent with the higher levels of Vcam1 in LT-HSC (Figure 3). Sox6 has mainly been implicated in cartilage formation as a downstream mediator of Bmp signaling [67], but it can also regulate transcription of the cell adhesion molecules N- and E-cadherin and of Wnt1 [68]. By affecting these regulators of HSC function, Sox6 could play important roles in HSC biology. However, *Sox6* null mice have no obvious hematopoietic phenotype, suggesting the presence of redundant factors or mechanisms. Hmgb3 has previously been shown to be expressed by HSC, and its downregulation was important for differentiation into myeloid and lymphoid but not erythroid lineages [69]. The lower levels of Hmgb3, Gata1, Gata2, and Nfe2 transcripts may contribute to attenuated erythroid and megakaryocyte potential of MPP compared to LT-HSC.

Nuclear factors upregulated in MPP include Ddx4, Hoxa9, Satb1, Atrx, Notch1, and Tcf12. Tcf12 may play a role in the proliferation of neural stem and progenitor cells [70], and its upregulation in MPP could indicate a similar role in hematopoiesis. *Hoxa9* and several other *Hox* genes have important roles in hematopoiesis, and several Hox members are involved in chromosomal translocations in leukemogenesis. *Hoxa9* null mice have significant reductions of both myeloid and lymphoid progenitor populations [71], and overexpression of Hoxa9 can expand the HSC pool [72], indicating that Hoxa9 is a regulator of hematopoietic stem and progenitor cell pool size. The expression of Ddx4, a homolog of *Drosophila* Vasa, in MPP is surprising as it has been shown previously to be selectively expressed in the germline [73]. Ddx4 has RNA helicase activity and is involved in RNA-related processes such as translation initiation, splicing, and nucleosome assembly. Upregulation of Ddx4 in MPP may reflect increased or selective translational activity compared to LT-HSC.

Although Notch1 has been implicated in HSC self-renewal [9], transcript levels of Notch1 increase as LT-HSC differentiate to MPP. Notch1, as well as the chromatin-remodeling protein Satb1, have roles in lymphoid development and cell fate decisions. Mutations of another chromatin remodeling factor upregulated in MPP, Atrx, are associated with mental retardation and α-thalassemia. Atrx may play roles in spindle organization and chromosome alignment during cell division [74], in addition to modifying chromatin structure to regulate transcription. As several transcriptional activators and repressors have been implicated both in HSC self-renewal and as master regulators of hematopoiesis, the role of transcriptional regulators not previously known to be expressed by HSC, such as Dachshund, will be interesting to examine, and genes regulated by a potential Dach/Ski/Eya2 complex may play important roles in LT-HSC fate decisions.

## Discussion

**Figure 5:**
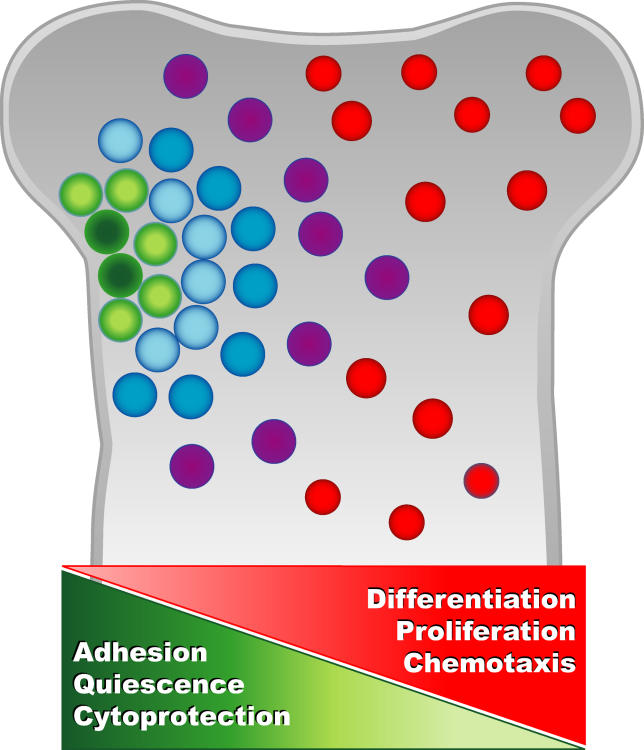
Schematic of Biological Processes that Gradually Decline or Increase with LT-HSC Differentiation Based on the Relative Transcript Levels Presented in this Report Green circles represent LT-HSC, and color gradients from green to red represent increasingly mature progeny.

Here, we have analyzed in detail the difference in transcriptional profiles between LT-HSC and two closely related isolatable progeny during steady-state hematopoiesis. This analysis revealed numerous transcripts not previously known to be expressed by HSC. Directly comparing highly purified LT-HSC to closely related progeny as opposed to heterogeneous cell populations or cells of nonhematopoietic lineages likely enhanced the sensitivity of the analysis. It is important to note that our analyses focused on differential expression and therefore did not seek to identify genes that are important for HSC biology but are not differentially expressed at the transcript level among LT-HSC, ST-HSC, and MPP. Posttranscriptional regulation and interaction with differentially expressed molecules may regulate the activity of such proteins. The differentially regulated transcripts we identified likely play important roles in early hematopoietic decisions, including self-renewal and quiescence of LT-HSC, and differentiation into the more proliferative ST-HSC and MPP. Collectively, these data support previous evidence suggesting that processes such as quiescence, adhesion, and cytoprotection are particularly important for LT-HSC integrity, while differentiation, proliferation, and chemotaxis are increasingly important for the immediate progeny (Figure 5). Novel candidates regulating these processes provide a model that will guide functional studies.

As shown in Figure 3, our analysis also led to the identification of new differentially expressed cell surface markers. It is important to note that there are multiple ways to isolate HSC and multipotent progenitors [1,16,18,75–79]. Although populations isolated by alternative methods show significant overlap with the subsets described here, there is likely even more complexity beyond the ones reported here. For example, functionally distinct multipotent progenitors can be separated based on CD4 and Mac1 expression [76], and as these subsets express low levels of Thy1.1, they are likely more primitive than the Thy1.1-negative MPP described here. It is possible that the MPP we identified by Flk2 expression are determined to respond to inflammatory stimuli. In addition, since LT-HSC as defined here can be divided into two fractions by at least two different surface markers (Slamf1 and CD34), we anticipate that these and other novel antigens will be useful in the ongoing refinement of the relationship between hematopoietic cell types and will increase our understanding of cell fate decisions.

The relative quiescence of LT-HSC and stem cells of nonhematopoietic lineages likely protects these cells from exposure to reactive oxygen species and toxic metabolites that could lead to DNA damage. Avoiding inheritable damage would be particularly important for stem cells, since they give rise to billions of mature cells throughout the life of the host. Insults to the hematopoietic system could be minimized if LT-HSC were resistant to cellular damage and then capable of replacing the shorter-lived mature cells. In addition to quiescence, the upregulation of transcripts associated with cytoprotection such as p53, Ches1, Sesn1, catalase, and Abcg2 in LT-HSC may play important roles in maintaining the integrity of LT-HSC.

Importantly, Abcg2 and other ATP-binding cassette transporters are associated with multidrug resistance of cancer cells, and an important goal in cancer biology is to understand the differences and similarities between normal stem cells and cancer stem cells [80]. We recently identified the leukemia-initiating cell fraction in a mouse model resembling human chronic myelogenous leukemia (CML), where the transplantable leukemia was contained in the phenotypic LT-HSC compartment [81]. To understand the molecular events leading to CML, we compared the expression profile of these leukemic HSC to normal HSC. Many of the transcripts preferentially expressed by normal LT-HSC are downregulated in the leukemic HSC (E. Passegué, E. C. F., and I. L. W., unpublished data), emphasizing the importance of these genes in the regulation of normal stem cells. We have also noted that in human CML the leukemic stem cell is at the HSC stage during the early, more indolent form of the disease, but when myeloid blast crisis emerges the candidate leukemic stem cell is found in a population of normally non-self-renewing cells [82]. In addition to clarifying the events leading to neoplastic transformation, understanding the similarities and differences between normal and oncogenic self-renewing cells will help specific therapeutic targeting of cancer-initiating cells while sparing normal HSC.

It is intriguing that many of the differentially regulated transcripts represent components of functional units such as receptor-ligand pairs and protein complexes as exemplified by the cell junction category (see Figure 4). Although some of these molecules can be found at adherens junctions, several are specifically associated with tight and gap junctions. Roles for these types of junctions have not been described in HSC; thus, determining their function will provide novel insights to HSC biology. We are currently assessing the functional role of Esam1 in hematopoiesis and the use of this antigen as a highly selective marker for LT-HSC. The details of those studies will be presented elsewhere.

The bone marrow microenvironment is crucial for adult HSC regulation. However, in view of the number of differentially expressed matrix molecules (i.e., Biglycan, ApoE, collagens), homotypic-interacting proteins (i.e., Esam1, Mllt4, Slamf1, claudins, and connexins), and ligand-receptor pairs (Angpt1:Tek, Slitl2:Robo4, Ccl3:Ccr5, and IL1r1:IL1b) these cell populations seem surprisingly self-sufficient in controlling their fates. The importance of these types of interactions for HSC function has been demonstrated previously, for example between integrin α4β1 and Vcam1, and between c-kit and SLF [14,83]. It should be noted, however, that the simultaneous expression of interacting molecules by the same cell presents cell biological puzzles concerning the prevention or use of intermolecular interactions and potential signaling during processing through the endoplasmic reticulum, the Golgi, and post-Golgi vesicles. Although other factors and cell types likely play important roles, LT-HSC might be capable of modifying their immediate environment by secreting matrix molecules as well as ligands for surface receptors. The abundance of homotypic-interacting surface molecules on LT-HSC suggests that LT-HSC interact with cells expressing at least some of the same molecules. Thus, it seems possible that HSC interact with each other and that they may reside in localized clusters in the bone marrow. The difficulty of identifying functional HSC in their native environment makes it even more challenging to determine what cell types interact with HSC in situ. Our identification of these novel HSC surface proteins could support current evidence for interactions between HSC and osteoblasts [10,11,62,84] and facilitate the search for other cell types capable of interacting with LT-HSC. In this regard, the expression of some of the same molecules upregulated by LT-HSC (biglycan, Gjb5, Slitl2) by AFT024 [85] may account for this stromal line's capability of supporting LT-HSC in culture for several weeks [86].

Although interaction with the niche may be a prerequisite for both HSC quiescence and self-renewal, these two processes might be developmental choices since a truly quiescent cell is not dividing. Thus, self-renewal requires signals to proliferate, in combination with prevention of apoptosis and differentiation. As many cell types are capable of self-renewal, although for a limited time or number of divisions, extended self-renewal may be regulated to a greater extent by inhibition of differentiation than by active promotion of self-renewal. The two main mechanisms regulating LT-HSC properties could be location and unresponsiveness to mitogenic and differentiation signals. Proper localization of LT-HSC within the bone marrow would be mediated by adhesion molecules preferentially expressed by LT-HSC. This niche might be characterized by high levels of molecules promoting quiescence and preventing differentiation, and by relatively low levels of signals favoring proliferation and differentiation. LT-HSC may contribute to the establishment of such gradients by expressing ligands, protease inhibitors, and extracellular matrix molecules, all of which would help retain the LT-HSC in the niche, hiding from toxic metabolites and mitogenic and differentiation signals (see Figure 5). We propose that at the appropriate time, HSC are induced to express relatively high levels of cyclin D3, which may enable them to overcome the antiproliferative signals and allow LT-HSC to undergo self-renewing divisions in the niche. More extensive proliferation and differentiation may require cells to move out of the niche. This might be facilitated by enzymes that inhibit cell adhesion, such as heparanase and metalloproteinases. Because MPP express higher levels of these enzymes, as well as cytokines associated with more differentiated cell types, some of this regulation could be cell autonomous.

The second proposed major regulator of LT-HSC, relative unresponsiveness to proliferation and differentiation signals, could be due to lack of receptors and signaling molecules mediating such signals and by high levels of cell cycle inhibitors. In contrast, ST-HSC and MPP may be poised to move down a differentiation pathway, in part by their inability to adhere to the niche, and in part by being more responsive to differentiation and mitogenic signals (Figure 5). Differentially expressed genes will cause two different cells exposed to the same signals to respond differently. For example, HSC with long-term repopulating ability circulate in the blood under normal homeostatic conditions [33], upon transplantation, and when induced to mobilize. Thus, leaving the niche does not induce irreversible differentiation in all cases. It is possible that passage through the blood and reentry into the bone marrow allows for stochastic relocation of cells to different kinds of available niches, some that direct the cells to regain quiescence, others to self-renew, or still others to differentiate. Such a model also provides a means for LT-HSC to sense and respond to homeostatic changes in the hematopoietic system more easily than if they remained in the niche. Quiescence itself may contribute to the unresponsiveness of LT-HSC, since differentiation requires proliferation. In addition, adhesion reinforces quiescence and vice versa, creating a positive feedback loop.

As the definition of HSC currently depends mainly on transplantation into irradiated hosts followed by assessment of long-term multilineage readout, it is difficult to determine to what extent an endogenous cell with a ST-HSC or MPP cell surface phenotype self-renew normally as opposed to upon transplantation. In addition, long-term engraftment upon transplantation is dependent on cell cycle status, with quiescent cells having higher engraftment potential (E. Passegué, A. J. Wagers, and I. L. W., unpublished data) [87,88]. The current transplantation-based definition of LT-HSC is therefore directly tied to quiescence. When assayed experimentally, long-term engraftment potential may thus be more a measure of a cell's ability to find and then remain in an environment supporting self-renewal divisions than a measure of greater intrinsic ability to self-renew. Given this perspective, it is not surprising to find an abundance of cell–cell and cell–matrix molecules on the surface of LT-HSC. At this point, we can only speculate on the potential implications of the novel LT-HSC-enriched transcripts. However, the number and variety of adhesion molecules we have identified as upregulated on LT-HSC support an important role for such molecules in LT-HSC function. In addition, the types of cell surface molecules identified in this report allow for dynamic regulation, since several receptors are regulated by soluble ligands and proteases, and recent literature has provided convincing evidence for the importance of such mechanisms in HSC function. Arai et al. proposed that Tek actively promotes adhesion and quiescence when bound by its ligand angiopoietin1 [12], and Mmps facilitate HSC differentiation by facilitating exit from the niche [13]. Our data support these reports and reveal numerous additional candidates that are likely to participate in dynamically regulated niche-cell interactions. Clearly, LT-HSC express genes that enable them to participate in complex cell–cell and cell–matrix interactions that direct their function and developmental decisions. These and other differentially expressed transcripts allowed us to generate a model that will guide our current functional approaches to understand HSC biology.

## Materials and Methods

### Isolation of cells.

LT-HSC (Lin^−^/c-kit^+^/Sca1^+^/Thy1.1^lo^/Flk2^−^), ST-HSC (Lin^−^/c-kit^+^/Sca1^+^/Thy1.1^lo^/Flk2^+^), and MPP (Lin^−^/c-kit^+^/Sca1^+^/Thy1.1^−^/Flk2^+^) were isolated from bone marrow of 8- to 12-wk-old BA mice by double-sorting on a modified FACS Vantage (Becton-Dickinson, Mountain View, California, United States) as described [16]. Appropriate functional readout was confirmed by transplantation into lethally irradiated hosts (unpublished data).

### RNA isolation, amplification, and labeling for array analysis.

Cells from individual isolations were pooled into 30,000- to 50,000-cell aliquots and RNA was extracted by Trizol. DNaseI treated RNA was amplified in two rounds by Arcturus RNA amplification kit following instructions from the manufacturer (Arcturus, Mountain View, California, United States). Amplified RNA (2.5 μg) was reverse transcribed with amino-allyl labeled deoxynucleotides (Ambion, Austin, Texas, United States) and dye-labeled by incubation with Cy3 or Cy5 for 1 h (http://cmgm.stanford.edu/pbrown/protocols/index.html). Probes were combined for the appropriate comparisons, purified with QiaQuick PCR purification kit (Qiagen, Valencia, California, United States) and eluted twice with 30 μl of EB. In each experiment, all probes were labeled by both Cy3 and Cy5 in separate reactions and combined with its comparison partner labeled in the other color in “dye-swap” experiments to eliminate bias due to differences in dye labeling efficiency. Volumes were adjusted to 28 μl by vacuum centrifugation and brought to 35 μl with a final concentration of 0.3% SDS and 3.4× SSC.

### cDNA array hybridization.

Competitive hybridizations on Stanford Microarray Facility 42k mouse cDNA arrays (http://www.microarray.org) were performed in three independent experiments, each time in duplicate to accommodate “dye swaps,” for a total of six hybridizations for each pairwise comparison. Combined probes were denatured and layered on Stanford cDNA microarray slides, coverslipped, and placed in sealed, humidified chambers in a 65 °C waterbath overnight (12–16 h). Slides were washed in 0.5× SSC with 0.01%SDS for 5 min and 0.06× SSC for 2 min. Slides were scanned with a Genepix 4000A scanner using GenepixPro software. Care was taken to match Cy3 and Cy5 emission spectra during scanning.

### Data analysis.

Scanned images were gridded with GenepixPro. “Bad” or missing spots were flagged by the software as well as visually. Resulting files were submitted to the Stanford Microarray Database (http://genome-www.stanford.edu/microarray) to assign identity to each spot and to normalize Cy3/Cy5 relative intensities for each slide. The log_2_ net intensity ratios were print-tip normalized (see below). Type I hybridizations directly comparing two different populations on the same array and therefore under exactly the same conditions enables a highly sensitive detection of differences between two populations without normalizing to a common reference that may vary from sample to sample. To further enable detection of the small, but potentially biologically highly significant, differences between the small developmental steps our three populations represent, no filters for intensity levels or spot size and shape were applied. Instead, our most stringent criterion was reproducibility. Six hybridizations in three independent experiments were performed for each pairwise comparison, and spots had to be present (not flagged as undetectable or bad) on at least five out of the six pairwise comparisons. Spots passing this criterion were analyzed for statistically significant differences using Significance Analysis of Microarrays (SAM; http://www-stat.stanford.edu/~tibs/SAM/) [89]. Because some genes were represented by multiple spots, each list was filtered by Unigene ID, clone ID, and GenBank accession number to determine the number of unique genes in each list. The number of spots and transcripts resulting from these analyses are summarized in Table 1. Significantly differentially regulated genes as defined by SAM at a false discovery rate of 10% are listed in Tables S1–S6.

### Print tip normalization.

Chip- or dye-specific biases were corrected for by applying print-tip loess using the BioConductor R package to all log-ratios. First, spots with intensity signals indistinguishable from background were filtered out. To achieve high signal-to-noise, any spot with average foreground intensity within 10% of the average background level was flagged as absent. Within each print-tip group, the mean and standard deviation of the log-ratios were computed within a window of values for the log-product. The log-ratios within this window were then centered and scaled using the estimated mean and standard deviation. This was applied to each window and repeated across all print tips.

### Gene Ontology (GO) analysis.

GO classification and statistics were performed using GOstat (http://gostat.wehi.edu.au/) and eGOn (http://nova2.idi.ntnu.no/egon/) with similar results. Genes upregulated in the less differentiated cell type of each comparison were combined to make an “undifferentiated” list (genes up in LT-HSC compared to either ST-HSC or MPP and genes up in ST-HSC compared to MPP). The “differentiated” list was made by combining genes upregulated in the more differentiated cell type (genes up in MPP versus either ST-HSC or LT-HSC, and genes up in ST-HSC versus LT-HSC). Similar results were obtained when genes upregulated and downregulated in the “LTvsMPP” comparison were used as the only input.

### Real-time RT-PCR.

Total RNA was isolated using Trizol reagent (Invitrogen, Carlsbad, California, United States) from equal numbers of purified LT-HSC, ST-HSC, and MPP (typically 8,000−10,000 cells), digested with DNaseI, and used for reverse transcription according to the manufacturer's instructions (SuperScript II kit, Invitrogen). For qRT-PCR analysis, primer sequences were designed using Primer Express software (Applied Biosystems, Foster City, California, United States) and are available upon request. PCR amplification was performed in triplicate in a 10 μl final volume containing cDNA corresponding to approximately 200 cells, and 1× SYBR Green PCR buffer, 2 mM magnesium chloride, 0.5 mM dNTP mix with dUTP, 1 μM of each primer, 0.1 U AmpErase UNG, and 0.25 U AmpliTaq Gold. All reactions were performed in an ABI-7000 sequence detection system (Applied Biosystems) at 50 °C for 2 min and 95 °C for 10 min followed by 40 cycles at 95 °C for 15 s and 60 °C for 1 min. For each sample, expression of the *HPRT* gene was used to normalize the amount of the investigated transcript.

### Cell surface protein levels by flow cytometry.

Cell surface protein expression was assessed by staining for LT-HSC, ST-HSC, and MPP with antibodies as described above, except an additional color was used (Cy7PE) to accommodate the additional antigen. Antibodies used were αVCAM1-biotin (eBioscience, San Diego, California, United States; clone #429), αCD9-biotin (BD Pharmingen, San Diego, California, United States; #KMC8), αSdc1-biotin (BD Pharmingen; #281–2), αESAM1 (clones 4G8 and 3C10) [25] conjugated to Alexa488, αSlamf1-PE (BioLegend, San Diego, California, United States; #TC15-12F12.2), αCD34-FITC (BD Pharmingen; #RAM34), αTek-biotin (eBioscience; #Tek4), and αJam2-FITC (SeroTec, Kidlington, Oxford, United Kingdom; #CRAM-18 F26).

Large-scale cDNA microarrays similar to the ones used for this study are available to researchers at not-for-profit research institutions by contacting the Stanford Functional Genomics Facility, CCSR 4256, 269 Campus Drive, Stanford, CA 94305-5177, United States, or by visiting http://www.microarray.org.

## Supporting Information

Figure S1 Quantitative RT-PCR of a Subset of Transcripts Identified as Differentially Regulated by Array Analysis Transcript levels are plotted as fold difference relative to LT-HSC after normalization to HPRT transcript levels. Blue bars represent ST-HSC and red bars represent MPP. Fold difference for genes off scale: Grb10, 10×; Nupr1, 29×; Sdc2, 62×; and Tgm2, 37×.
(554 KB PDF)

Figure S2 Chromosomal Distribution of Genes Differentially Regulated Between LT-HSC and MPP The distribution of genes that are differentially regulated between LT-HSC (green) and MPP (red) were normalized to total number of differentially regulated genes with known chromosomal location for each population.
(71 KB PDF)

Table S1 Transcripts Upregulated in ST-HSC Compared to LT-HSC (181 KB XLS)

Table S2 Transcripts Upregulated in LT-HSC Compared to ST-HSC (99 KB XLS)

Table S3 Transcripts Upregulated in MPP Compared to LT-HSC (160 KB XLS)

Table S4 Transcripts Upregulated in LT-HSC Compared to MPP (260 KB XLS)

Table S5 Transcripts Upregulated in MPP Compared to ST-HSC (18 KB XLS)

Table S6 Transcripts Upregulated in ST-HSC Compared to MPP (53 KB XLS)

Table S7 Transcripts Upregulated in LT-HSC Mapped to the X Chromosome (62 KB PDF)

Table S8 Additional References (52 KB XLS)

### Accession Numbers

The LocusLink accession numbers (LLIDs) (http://www.ncbi.nlm.nih.gov/projects/LocusLink/) of the proteins discussed in this paper are 4a2 (12827), Abcg2 (26357), Aldh1a1 (11668), Aldh7a1 (110695), angiopoietin 1 (11600), ApoE (11816), App (11820), Atrx (22589), biglycan (12111), Blnk (17060), Calsyntenin1 (65945), catalase (12359), Ccl3 (20302), Ccl4 (20304), Ccl9 (20308), Ccr5 (12774), CD34 (12490), CD63 (12512), CD9 (12527), Cenpe (229841), Cenph (26886), Ches1 (71375), Claudin12 (64945), Claudin22 (75677), c-myc (17869), collagens type 4a1 (12826), cortactin (13043), cyclin A2 (12428), cyclin B1 (268697), cyclin B2 (12442), cyclin D1 (12443), cyclin D3 (12445), Cyp2j6 (13110), Dachshund1 (13134), Dag1/dystroglycan (13138), Ddx4 (13206), Emilin1 (100952), Epb4.1 (269587), ESAM1 (69524), Eya2 (14049), Fgd1 (14163), Fstl1 (14314), Gata1 (14460), Gata2 (14461), Gelsolin (227753), Gja1 (14609), Gjb5 (14622), Grb10 (14783), Grb2 (14784), Gsta1 (14857), Gstk1 (76263), Gstm1 (14862), heparanase (15442), Hmgb3 (15354), Hoxa5 (15402), Hoxa9 (15405), Hoxb5 (15413), Ier5 (15939), Igh6 (16019), IL1β (16176), IL10rα (16154), IL10rβ (16155), IL17r (16172), IL1r1 (16177), Itga6 (16403), Jam1/F11R (16456), Jam2 (67374), Jam3 (83964), Ki67 (17345), Kif22 (110033), Kif23 (71819), Kif4 (16571), Kifc5a (94116), Lims1 (110829), Map17 (67182), Mllt4 (17356), Mlp (17357), Mmp15 (17388), Mmp16 (17389), Mmp2 (17390), Mpeg1 (17476), necdin (17984), Neogenin (18007), Nfe2 (18022), Notch1 (18128), Notch1 (18128), p53 (22059), p57/Cdkn1c (12577), Pbx1 (18514), Pbx3 (18516), Robo4 (74144), sarcoglycanE (20392), Satb1 (20230), Sdc1 (20969), Sdc2 (15529), Sesn1 (140742), Ski (20481), Slamf1 (27218), Slit-like2 (246154), Smc1l1 (24061), Smc2l1 (14211), Smc4l1 (70099), Sox18 (20672), Sox6 (20679), Spag5 (54141), Srgap2 (14270), Tcf12 (21406), Tek (21687), Tgm2 (21817), Timp2 (21858), Timp3 (21859), Tjp1/ZO-1 (21872), Top2a (21973), and VCAM1 (22329).

## Abbreviations

App: amyloid beta precursor protein
CML: chronic myelogenous leukemia
ECM: extracellular matrix
EST: expressed sequence tag
GO: Gene Ontology
HSC: hematopoietic stem cell(s)
LT: long-term
Mmp: matrix metalloproteinase
MPP: multipotent progenitor(s)
SAM: Significance Analysis of Microarrays
ST: short-term
