# Supplementary material for: Differential Expression of Novel Potential Regulators in Hematopoietic Stem Cells
Source: PLoS Genet. 2005 Sep 2;1(3):e28. doi: 10.1371/journal.pgen.0010028 (PMC1200425; doi:10.1371/journal.pgen.0010028)

Forsberg Supplemental Figure 1

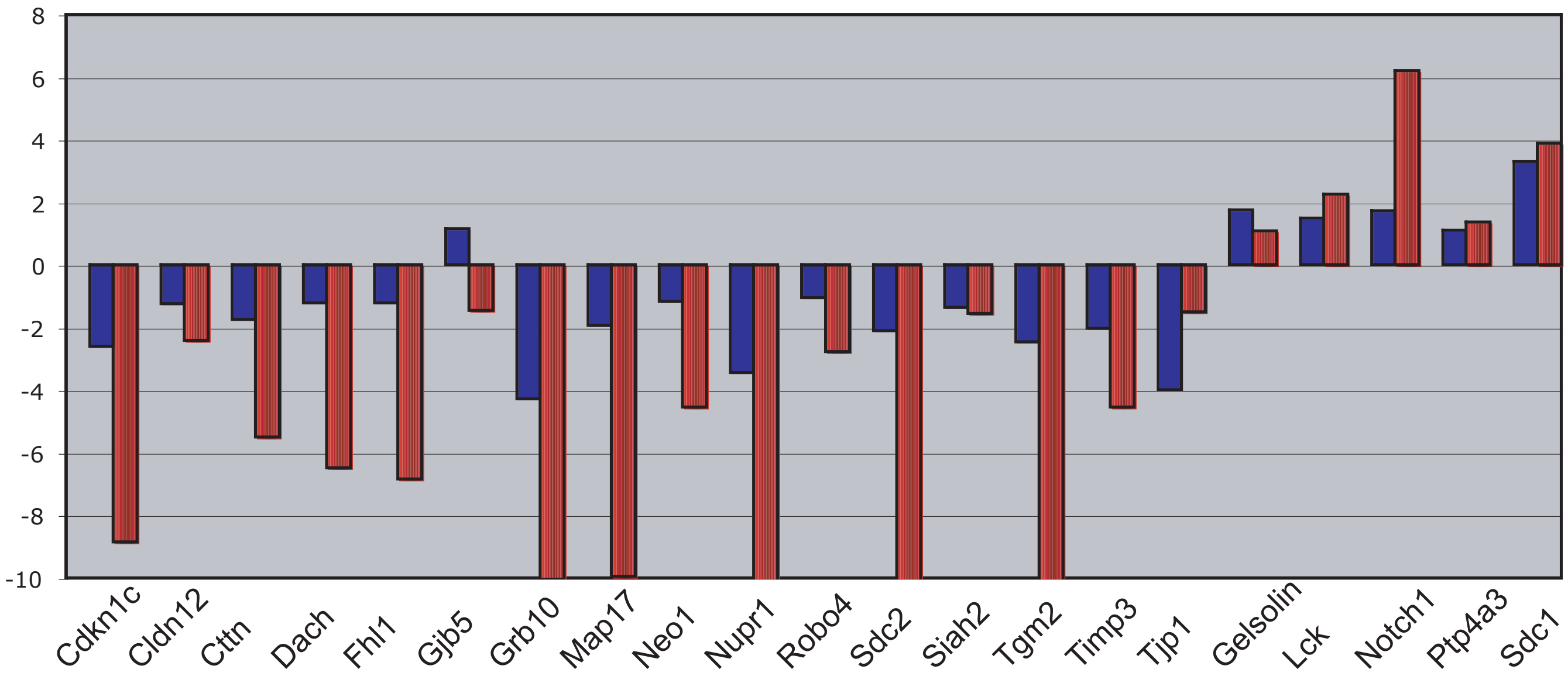

Supplement: Supplementary file 1 [file pgen.0010028.sg001.pdf]
