# Supplementary material for: Differential Expression of Novel Potential Regulators in Hematopoietic Stem Cells
Source: PLoS Genet. 2005 Sep 2;1(3):e28. doi: 10.1371/journal.pgen.0010028 (PMC1200425; doi:10.1371/journal.pgen.0010028)
Supplement: Supplementary file 3 [file pgen.0010028.st007.pdf]

Forsberg Supplemental Table 7

Symbol	Name	UGID	Cytoband
Itm2a	Integral membrane protein 2A	Mm.193	X
Fgd1	FYVE, RhoGEF and PH domain containing 1	Mm.219461	X
Rex3	Reduced expression 3	Mm.14768	X
Bgn	Biglycan	Mm.2608	X
Gata1	GATA binding protein 1	Mm.335973	X
Mid1ip1	Mid1 interacting protein 1 (gastrulation specific G12-like (zebrafish))	Mm.29429	X A1.1
Klhl13	kelch-like 13 (Drosophila)	Mm.224306	X A2
Mospd1	motile sperm domain containing 1	Mm.28236	X A4
Fhl1	four and a half LIM domains 1	Mm.3126	X A6-A7.1
Hmgb3	high mobility group box 3	Mm.336087	X A7.1
Pls3	plastin 3 (T-isoform)	Mm.28777	X A7.2-12
Pdk3	pyruvate dehydrogenase kinase, isoenzyme 3	Mm.12775	X C1
Dlgh3	discs, large homolog 3 (Drosophila)	Mm.4615	X C3
Magee1	melanoma antigen, family E, 1	Mm.24341	X D
3930402F23Rik	RIKEN cDNA 3930402F23 gene	Mm.182094	X E3
LOC406217	RIKEN cDNA 2410004M13 gene	Mm.336245	X E3
Psmd10	proteasome (prosome, macropain) 26S subunit, non-ATPase, 10	Mm.17640	X F1
Dsip1	delta sleep inducing peptide, immunoreactor	Mm.22216	X F1
Rnf128	ring finger protein 128	Mm.27764	X F1
AI316885	expressed sequence AI316885	Mm.30506	X F2
Lrch2	Leucine-rich repeats and calponin homology (CH) domain containing 2	Mm.30506	X F2
Maged2	melanoma antigen, family D, 2	Mm.22575	X F3
Smc1l1	SMC (structural maintenance of chromosomes 1)-like 1 (S. cerevisiae)	Mm.26412	X F3
